# The impact of preclinical education on operative and restorative skills in dental students: a survey-based evaluation

**DOI:** 10.1186/s12909-025-08252-x

**Published:** 2025-11-25

**Authors:** Elif Türkeş Başaran, Burcu Dikici, Gülşah Yenier Yurdagüven, Haktan Yurdagüven

**Affiliations:** 1 Department of Restorative Dentistry, Faculty of Dentistry, Yeditepe University, Bağdat St. No:238, İstanbul, Kadikoy 34728 Turkey; 2 Department of Restorative Dentistry, İstanbul Okan University, İstanbul, Turkey

**Keywords:** COVID-19, Clinical training, Student self-confidence, Preclinical training

## Abstract

**Aim:**

In response to the nationwide measures due to the COVID-19 pandemic, face-to-face preclinical dental education transitioned to a hybrid format. This survey aimed to assess the impact of hybrid education on dental students’ transition to clinical practice and to evaluate the efficacy and sustainability of preclinical education delivered in a hybrid model.

**Methods:**

The multi-centered questionnaire survey was e-mailed to students who received preclinical education in dentistry faculties from 2018 to 2020 and subsequently proceeded to clinical education. Students who received preclinical education in face-to-face and hybrid formats were included. The survey examined how preclinical education affects restorative clinical skills and students’ perceptions. It compared students with different levels of preclinical education using descriptive statistics and the Mann-Whitney U test for quantitative analysis (*p* < 0.05).

**Results:**

Analysis of responses from 341 participants revealed that students who received face-to-face preclinical education exhibited greater confidence in performing restorative procedures than students educated in a hybrid format. Nonetheless, both groups demonstrated a high level of self-assurance.

**Conclusion:**

This study highlights the challenges faced by students in hybrid learning and provides strategies to overcome them. With proper adjustments, hybrid learning can become a reliable and flexible component of dental education, maintaining continuity and quality even in challenging circumstances. The study also informs future curriculum planning by emphasizing the importance of preparedness for distance-based learning, flexibility in clinical scheduling, and early identification of gaps in clinical skill development.

**Supplementary Information:**

The online version contains supplementary material available at 10.1186/s12909-025-08252-x.

## Introduction

The COVID-19 pandemic, declared by the World Health Organization in March 2020, significantly impacted global education systems, including dental schools. Several behavioral and social measures, such as social distancing, were proposed and implemented to prevent and reduce viral spread. To follow social distancing guidelines and ensure student safety, universities worldwide quickly transitioned to remote learning [1, 2]. While theoretical courses were effectively adapted to online formats, preclinical dental education—crucial for developing students’ psychomotor skills—faced unique challenges. The suspension of hands-on training during the pandemic disrupted practical learning environments, prompting innovative solutions to maintain the continuity and quality of skill development [3, 4].

Online learning is not new; higher education has offered online courses since the mid-1990s, and technological advances have broadened synchronous and asynchronous modalities [5, 6]. Synchronous formats (e.g., live online lectures/webinars via Zoom or Google Meet) and asynchronous resources (e.g., recorded videos, virtual libraries) provide flexibility and access. However, shifting dental education completely online faced major challenges, especially for preclinical and clinical training, which rely heavily on hands-on practice and direct interaction with instructors [7, 8].

Preclinical dental education prepares students for clinical practice by developing essential psychomotor skills for operative and restorative procedures. This phase involves challenges such as limited hands-on opportunities, individual variations in learning curves, and the need for consistent, high-quality feedback. The pandemic highlighted these limitations, emphasizing the need for adaptable training methods.

Traditionally, preclinical training relies on physical models to develop manual dexterity and competence in controlled environments. Instructors use anatomical models, instructional videos, and live demonstrations to enhance learning. Simulation-based training and virtual reality (VR) technologies have emerged as promising alternatives, offering risk-free practice environments with personalized feedback and objective performance measurement. Despite their potential, these technologies remain inconsistently integrated into dental curricula, with traditional model-based training still dominating [9–11].

Dental education typically includes three main parts: theoretical instruction, preclinical training, and clinical education. While the theoretical parts were easily shifted to remote formats during the pandemic, preclinical courses—especially those needing direct instructor supervision and hands-on practice—were hard to adapt. These courses require face-to-face interaction and manual work on models, which makes them unsuitable for fully online delivery [10, 12].

Students’ self-esteem and confidence are essential components of the educational process and play a critical role in fostering a positive learning environment. These psychological attributes not only facilitate knowledge acquisition but also provide a sense of security in clinical roles, supporting students’ adaptation to professional responsibilities. Confidence enables students to approach new challenges with competence, while healthy self-esteem encourages active participation, critical discussions, and inquiry in various learning scenarios [13]. Conversely, low self-esteem or a lack of confidence can impede students’ full engagement in the learning process and hinder their clinical development. In dental education, where the integration of clinical reasoning, psychomotor skills, and theoretical knowledge is paramount, these psychological factors significantly influence the formation of students’ professional identity and their preparedness for clinical practice [14].

Although many reports have highlighted pandemic-related changes in dental education, there is limited evidence directly connecting modified preclinical instruction to students’ perceived clinical preparedness. In this study, student self-assessments served as perception-based indicators of competence and educational impact, comparing confidence, engagement, and perceived learning outcomes across different instructional models. Accordingly, this study aimed to evaluate the impact of hybrid preclinical education on students’ transition to clinical practice, focusing on perceived effectiveness and sustainability. The null hypothesis (H_0_) posits that the mode of preclinical instruction—face-to-face versus hybrid—does not significantly affect students’ clinical readiness (and performance if objectively assessed).

## Methods

This is a multi-centered cross-sectional observational study using an electronically distributed questionnaire created with Google Forms on a population of 400 undergraduate students who received preclinical education between 2018 and 2020 at Yeditepe University and İstanbul Okan University Faculty of Dentistry. The survey remained open from April 5th to April 30th, 2020. A total of 341 students participated, with no exclusion criteria applied (response rate ≈ 85%).

The study received approval from the Ethics Committee of Yeditepe University Faculty of Dentistry (No: 202203Y0210). The study was conducted in accordance with relevant ethical guidelines, including the principles outlined in the Declaration of Helsinki. Before starting the survey, informed consent was obtained electronically and participant details were provided. Participants could leave at any time, and their participation was completely voluntary and anonymous.

A total of 182 undergraduate students took part in a preclinical restorative course during the COVID-19 pandemic. During campus closures, preclinical sessions were conducted live via Google Meet. Instructors demonstrated each procedure at the beginning of sessions, observed students step-by-step, and offered real-time feedback. Students practiced on take-home training models, supported by instructional videos shared for self-practice. Attendance was tracked. Assignments were submitted through email or Google Drive and graded by instructors. When limited campus access was allowed, students completed essential hands-on components in small groups to follow distancing and capacity restrictions. Outside mandatory sessions, access to preclinical laboratories was restricted.

Students who completed their preclinical education began clinical training the following year. A questionnaire was created to assess how well undergraduate dentistry students perceived their readiness during clinical practice based on their preclinical training. The questionnaire consisted of two parts: Part A included four questions about participants’ demographic details, while Part B contained 16 questions covering the clinical stages necessary for students to evaluate their clinical competence. Responses were based on a five-point Likert scale, ranging from 1 (strongly disagree) to 5 (strongly agree). This questionnaire was specifically developed by the authors for this study. *An English version is provided in Appendix 1.*

To ensure clarity and reliability, a pilot test was conducted with 10 individuals who met the inclusion criteria and the internal consistency was evaluated using Cronbach’s alpha, which resulted in a value of 0.949. The questionnaire was only modified semantically in response to pilot study comments. Academic representatives chosen by the individual deans disseminated the link to students for whom approval from the deans of academic institutions had been secured.

Participants read study information and provided informed consent online before performing the anonymous survey. Only students who received preclinical education between 2018 and 2020 were included in the study. Students who received preclinical education other than these years were not included in the study. The sample size was calculated using the G*Power program, with a 95% confidence level (1-α), 95% test power (1-β), and an effect size of w = 0.44, determining the minimum required sample size to be at least 133 [15].

The IBM SPSS Statistics 22 software was used for statistical analysis to assess the study’s results. The Shapiro-Wilk and Kolmogorov-Smirnov tests were used to evaluate the parameters’ normality, and the results showed that they did not exhibit a normal distribution. The Mann-Whitney U test was used to compare quantitative data between the hybrid and face-to-face learning groups. Descriptive statistical techniques (mean, standard deviation, and frequency) were utilized while assessing the study data. The significance level was set at *p* < 0.05.

## Results

A total of 341 students participated in this study, of which 159 (46.6%) were preclinical students who were educated face-to-face and the remaining 182 (53.4%) students took hybrid education. In addition, 236 students (69.2%) were female, while the remaining 105 students (30.8%) were male. The mean age was between 20 and 28 (23 ± 1.56). The demographic details of the students are summarized in Table 1.

**Table 1:** Distribution of general information

		*n*	%
Gender	Male	105	30,8
	Female	236	69.2
Grade	1	54	15.8
	2	122	35.8
	3	84	24.6
	4	47	13.8
	5	34	10.0
Education	Face to face	159	46.6
	Hybrid	182	53.4
Age		20–28	23 ± 1.56

Data on the impact of preclinical education type on clinical education are presented in Table 2.

**Table 2:** Evaluation of answers based on the method of education

		1	2	3	4	5	Total	
		*n* (%)	*n* (%)	*n* (%)	*n* (%)	*n* (%)	Mean ± SD (median)	*p*
I was able to detect occlusal and smooth surface caries	Face to face	2 (%1.3)	2 (%1.3)	6 (%3.8)	71 (%44.7)	78 (%49.1)	4.4 ± 0.7 (4)	0.000*
	Hybrid	4 (%2.2)	4 (%2.2)	22 (%12.1)	110 (%60.4)	42 (%23.1)	4 ± 0.8 (4)	
I was able to detect approximal surface caries	Face to face	2 (%1.3)	4 (%2.5)	13 (%8.2)	89 (%56)	51 (%32.1)	4.2 ± 0.8 (4)	0.000*
	Hybrid	6 (%3.3)	13 (%7.1)	39 (%21.4)	96 (%52.7)	28 (%15.4)	3.7 ± 0.9 (4)	
I used the hand instruments during treatment according to their intended purposes	Face to face	1 (%0.6)	2 (%1.3)	7 (%4.4)	64 (%40.3)	85 (%53.5)	4.4 ± 0.7 (5)	0.000*
	Hybrid	2 (%1.1)	5 (%2.7)	18 (%9.9)	110 (%60.4)	47 (%25.8)	4.1 ± 0.8 (4)	
I used rotary instruments and burs for their intended purposes during the treatment.	Face to face	1 (%0.6)	2 (%1.3)	7 (%4.4)	69 (%43.4)	80 (%50.3)	4.4 ± 0.7 (5)	0.000*
	Hybrid	2 (%1.1)	4 (%2.2)	18 (%9.9)	106 (%58.2)	52 (%28.6)	4.1 ± 0.7 (4)	
I removed caries effectively	Face to face	1 (%0.6)	5 (%3.1)	19 (%11.9)	72 (%45.3)	62 (%39)	4.2 ± 0.8 (4)	0.000*
	Hybrid	4 (%2.2)	16 (%8.8)	27 (%14.8)	102 (%56)	33 (%18.1)	3.8 ± 0.9 (4)	
I performed an ideal cavity preparation for an anterior composite resin restoration.	Face to face	5 (%3.1)	9 (%5.7)	19 (%11.9)	74 (%46.5)	52 (%32.7)	4 ± 1 (4)	0.000*
	Hybrid	7 (%3.8)	18 (%9.9)	39 (%21.4)	93 (%51.1)	25 (%13.7)	3.6 ± 1 (4)	
I performed an ideal cavity preparation for a posterior composite resin restoration.	Face to face	2 (%1.3)	3 (%1.9)	8 (%5)	83 (%52.2)	63 (%39.6)	4.3 ± 0.8 (4)	0.000*
	Hybrid	3 (%1.6)	7 (%3.8)	33 (%18.1)	105 (%57.7)	34 (%18.7)	3.9 ± 0.8 (4)	
I achieved ideal placement of the matrix and wedge in anterior interproximal cavities	Face to face	4 (%2.5)	10 (%6.3)	24 (%15.1)	71 (%44.7)	50 (%31,4)	4 ± 1 (4)	0.000*
	Hybrid	7 (%3.8)	19 (%10.4)	39 (%21.4)	86 (%47.3)	31 (%17)	3.6 ± 1 (4)	
I achieved ideal placement of the matrix and wedge in posterior interproximal cavities	Face to face	2 (%1.3)	4 (%2.5)	17 (%10.7)	70 (%44)	66 (%41,5)	4.2 ± 0.8 (4)	0.000*
	Hybrid	5 (%2.7)	9 (%4.9)	37 (%20.3)	94 (%51.6)	37 (%20,3)	3.8 ± 0.9 (4)	
I applied indirect and ergonomic working principles.	Face to face	7 (%4.4)	21 (%13.2)	54 (%34)	50 (%31.4)	27 (%17)	3.4 ± 1.1 (3)	0.019*
	Hybrid	19 (%10.4)	30 (%16.5)	55 (%30.2)	66 (%36.3)	12 (%6,6)	3.1 ± 1.1 (3)	
I applied liner materials according to their indications, guided by theoretical knowledge	Face to face	4 (%2.5)	8 (%5)	20 (%12.6)	79 (%49.7)	48 (%30,2)	4 ± 0.9 (4)	0.000*
	Hybrid	12 (%6.6)	27 (%14.8)	54 (%29.7)	69 (%37.9)	20 (%11)	3.3 ± 1.1 (3)	
I applied base materials in accordance with their indications, guided by theoretical knowledge	Face to face	4 (%2.5)	3 (%1.9)	27 (%17)	80 (%50.3)	45 (%28,3)	4 ± 0.9 (4)	0.000*
	Hybrid	10 (%5.5)	18 (%9.9)	30 (%16.5)	99 (%54.4)	25 (%13,7)	3.6 ± 1 (4)	
I accurately performed the clinical steps of adhesive system application	Face to face	1 (%0.6)	0 (%0)	10 (%6.3)	71 (%44.7)	77 (%48,4)	4.4 ± 0.7 (4)	0.000*
	Hybrid	5 (%2.7)	7 (%3.8)	15 (%8.2)	100 (%54.9)	55 (%30,2)	4.1 ± 0.9 (4)	
I used the layering technique to properly apply composite resins into the cavity	Face to face	1 (%0.6)	1 (%0.6)	7 (%4.4)	63 (%39.6)	87 (%54,7)	4.5 ± 0.7 (5)	0.000*
	Hybrid	4 (%2.2)	7 (%3.8)	18 (%9.9)	96 (%52.7)	57 (%31,3)	4.1 ± 0.9 (4)	
I completed the finishing and polishing procedures of the composite resin restorations ideally.	Face to face	1 (%0.6)	1 (%0.6)	6 (%3.8)	65 (%40.9)	86 (%54,1)	4.5 ± 0.7 (5)	0.000*
	Hybrid	3 (%1.6)	5 (%2.7)	18 (%9.9)	104 (%57.1)	52 (%28,6)	4.1 ± 0.8 (4)	
Ideally checked the occlusion and height at the end of the composite resin restorations	Face to face	1 (%0.6)	1 (%0.6)	7 (%4.4)	61 (%38.4)	89 (%56)	4.5 ± 0.7 (5)	0.000*
	Hybrid	10 (%5.5)	19 (%10.4)	23 (%12.6)	83 (%45.6)	47 (%25,8)	3.8 ± 1.1 (4)	

1 Strongly disagree, 2 Disagree, 3 Undecided, 4 Agree, 5 Strongly agree

Mann Whitney U test. *p<0.05

**Fig. 1:**
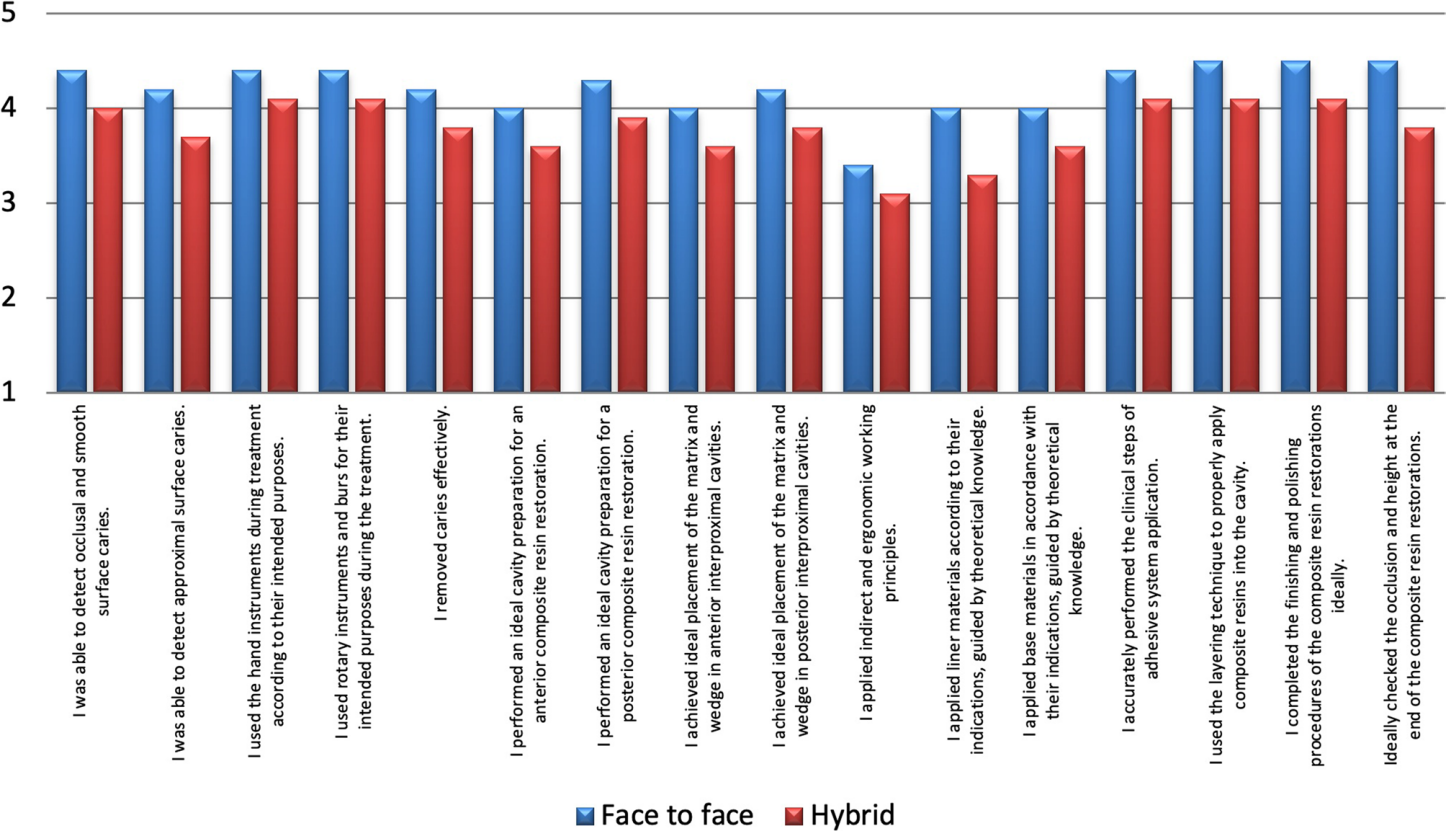
Comparison of student confidence and skills in restorative procedures

In both groups (“Face to Face” and “Hybrid”), participants generally expressed positive opinions. “Agree” and “Strongly agree” answers were dominant in many questions (Fig. 1).

The average scores of students receiving preclinical education face-to-face in terms of feeling competent in all the subjects were statistically significantly higher compared to the group receiving hybrid education (Fig. 1; *p* < 0.05).

**Fig. 2:**
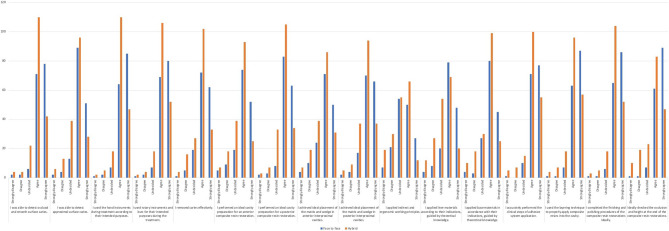
Analysis of student confidence and skills in restorative procedures across different preclinical education groups

The face-to-face group responded with high “Agree” and “Strongly Agree” rates on most questions. The hybrid group had more “Undecided” and “Disagree” responses. However, “Agree” and “Strongly Agree” responses were still prominent in many questions. The hybrid group was more diverse than the face-to-face group. Participants who received face-to-face training reported higher satisfaction overall than those who received hybrid training. There was more uncertainty (indecision) on some issues in the hybrid group (Fig. 2).

Students who received face-to-face education felt reasonably competent, especially in detecting occlusal and smooth surface caries and correctly using hand and rotary instruments during treatment. They predominantly marked the “strongly agree” option (Fig. 2).

Additionally, the face-to-face education group has predominantly marked the ‘strongly agree’ option, demonstrating high self-confidence regarding the application stages of adhesive systems, the layering technique of composite resins in cavities, and the finishing-polishing processes and occlusion control of restorations, which are stages of the restoration process. In the same statements, students who receive hybrid education have predominantly marked the “agree” option (Fig. 2).

Both education groups predominantly marked the ‘agree’ option regarding caries removal, cavity preparation in anterior and posterior approximal areas, placement of matrices and wedges in these areas, use of liner and base materials, and ergonomic working principles. Students who received hybrid preclinical education provided a high rate of “undecided” responses to these questions, except for the question about caries removal.

On the other hand, both groups expressed uncertainty about their ability to apply indirect and ergonomic working principles when transitioning to clinical practice, and they marked the “disagree” option at a higher rate than other applications (Fig. 2).

## Discussion

This multi-centered survey aimed to understand the effects of the education received by the Faculty of Dentistry students on the transition process to clinical practices and to determine the efficiency and sustainability of the preclinical education given as a hybrid over face-to-face education. Based on the results of this study, the null hypothesis stating that the type of preclinical education (face-to-face or hybrid) has no significant impact on clinical education was rejected.

Dental education requires both theoretical knowledge and manual skills. Over ten semesters of integrated dentistry studies, students are expected to gradually develop clinical competencies through practical training [16–18]. The COVID-19 pandemic disrupted traditional educational models, prompting dental schools in Turkey to close on March 15, 2020, with an immediate shift to distance learning to maintain continuity and safety [19, 20].

While remote learning introduced flexibility, self-paced study, and greater access to resources, its ability to teach manual skills remains limited [21–23]. The hands-on nature of dentistry, particularly for procedures requiring manual dexterity, cannot be fully replaced by online instruction [22, 23].

The hybrid model used during the pandemic aimed to close this gap. However, in this study, students who experienced hybrid preclinical education reported much lower self-confidence in complex procedures—such as cavity preparation in anterior and posterior approximal areas, matrix and wedge placement, use of liner and base materials, and ergonomic working principles—compared to students who received face-to-face training. Greater uncertainty and disagreement in the hybrid group likely come from limited hands-on experience and fewer opportunities for direct instructor feedback [24–28].

In contrast, the face-to-face group showed strong agreement or agreement in identifying occlusal and smooth surface caries and in properly using hand and rotary instruments, indicating that these more basic restorative tasks were less challenging for students who did not experience COVID-affected education. However, confidence decreased for more complex and skill-demanding tasks even among students trained in person, highlighting the inherent difficulty of mastering advanced manual procedures [25, 26].

An important strength of this study is its multi-center design, which improves external validity and generalizability. Different institutional and geographic settings allow for a wider application of the findings, and consistent results across centers strengthen reliability.

Turkyilmaz et al. [29] reported that e-learning has a significant positive effect on students’ understanding of both didactic and clinical components. While this emphasizes the potential of online education, it is important to acknowledge that, like traditional teaching methods, online education also has its benefits and downsides. The benefits of e-learning include the ability of platforms to track and analyze learning data, offering insights into how and when learning takes place. Additionally, students have greater access to information via mobile devices, and e-learning platforms can adapt dynamically to students’ needs, improving communication between students and instructors [9, 30, 31]. However, the limitations of online education should not be ignored. These include a lack of interpersonal communication, technical challenges such as device requirements, poor internet connectivity, and issues with time management, especially for students who struggle with self-regulation [32].

Furthermore, pandemic-related restrictions have forced many students to change their daily routines, which has affected their physical activity, sleep patterns, and social interactions. These changes have also impacted their mental well-being [33]. The shift to fully online education, being a new experience for many students, has influenced their behavior and learning efficiency, especially in adapting to new technologies and teaching methods. These findings offer insights into the strengths and weaknesses of online learning, especially compared to traditional face-to-face education, highlighting areas where improvements can be made.

Although students adapted well to the shift to online education, some challenges related to the loss of practical courses were evident, especially in areas requiring hands-on skills and direct patient interaction. This finding aligns with studies by Iosif et al. [34] and Hattar et al. [35], who reported no significant negative effects on students in similar situations. Similarly, Hung et al. [36] suggested that dental education could be improved by incorporating new learning methods during the COVID-19 pandemic, noting that students appeared to adapt well to the new technologies.

On the other hand, Cheng et al. [37] found that shifting to online instruction was feasible, although not ideal for dental schools with laboratory-based programs. Similarly, a study by Van Doren et al. [7] showed that while students valued the online teaching format, they did not believe it could replace the hands-on experience of direct patient care. Conversely, Sarialioglu Gungor et al. [38] discovered that senior students preferred online learning, noting that younger students might find it hard to understand and follow online lectures due to their limited background knowledge in the field.

In our study, it was observed that students who received hybrid education demonstrated lower self-confidence compared to those who participated in face-to-face education. This finding aligns with results reported in the literature regarding online education during the pandemic. For example, in three studies conducted by Büssing et al., it was reported that online teaching negatively impacted students’ stress perception, psychological well-being, and work satisfaction, particularly during the early stages of the pandemic when strict social restrictions were in place [39–41]. These findings provide valuable insights into how hybrid and online education methods shape students’ academic and psychological experiences. Designing educational methods that support students’ self-confidence and overall well-being should be a critical focus for future research.

Despite these challenges, online learning provides significant benefits, such as increased student participation and improved interaction [42]. This greater engagement is a major advantage of online platforms, but it is still important to recognize that certain aspects of education, particularly in fields like dentistry, cannot be fully replaced by online methods alone.

While online education can support the development of knowledge and theoretical understanding, clinical training programs are crucial for building students’ critical thinking and clinical reasoning skills. These programs simulate “real-world” scenarios and give students chances to practice clinical skills. However, electronic teaching—regardless of the technology used—cannot replace the invaluable experience of direct patient care and the hands-on learning opportunities provided by face-to-face clinical classes.

Although our institution does not currently use modern educational methods like virtual reality (VR) and haptics, the literature highlights their significant potential to improve dental students’ confidence and preclinical skills. For example, studies show that integrating VR and haptics into dental curricula can enhance motor skill training and complement existing simulation methods [12]. The feasibility and acceptance of haptic VR technology among dental students are linked to its utility and ease of use. This is a limitation of our study, as we could not assess the impact of these technologies. Future research should explore incorporating VR and haptics to maximize their potential benefits in dental education.

Clinical training programs focus on improving students’ critical thinking and clinical reasoning skills in various real-world situations, while also teaching and practicing clinical techniques. Electronic teaching, regardless of the technology used, cannot easily replace the hands-on patient care and in-person experience offered by clinical classes.

One potential concern about this study is the reliance on student self-assessment data for comparing models. However, we deliberately chose self-assessment as a key part of our evaluation process. Additionally, this is particularly important in dental education, where learners are expected to assume responsibility for clinical judgments gradually. Therefore, we believe including self-assessment in model comparisons is not only appropriate but also crucial for a comprehensive evaluation.

This study did not specifically investigate the factors affecting student self-confidence. However, the literature indicates that individual learning styles and the quality of educational materials significantly influence student confidence. For example, providing materials tailored to visual, auditory, or kinesthetic learning preferences can help students feel more capable. Likewise, educational materials that are current, accessible, and practice-oriented can enhance students’ confidence in their clinical skills [43]. Future research should examine these factors more thoroughly and aim to develop educational programs that address them more effectively.

Moreover, the formation of diagnoses and the creation of therapy plans are greatly shaped by theoretical knowledge, which has been consistently conveyed to all generations of students through the dedication of the professional staff.

## Limitations

This study has certain limitations. First, relying on self-assessment data can introduce subjectivity, as participants’ perceptions of their competence might not entirely match objective evaluations. Second, the study was conducted in two centers, which may limit the applicability of the findings to other institutions with different educational systems or resources. Finally, while the sample size is sufficient for statistical analysis, it might not encompass the full range of dental education experiences.

## Conclusion

This multi-centered survey study examines the perspectives of undergraduate dental students on hybrid learning during the COVID-19 pandemic, in comparison to face-to-face methods. The COVID-19 pandemic had a profound impact on academic personnel, dental students, and dental researchers, resulting in challenges in clinical work and practical training. While this study indicates that hybrid education can be effective for theoretical learning, it also reveals significant limitations in developing practical skills. These findings highlight the need for targeted adjustments and innovations to address these challenges. With appropriate refinements, hybrid learning has the potential to complement traditional face-to-face methods and ensure continuity in dental education during adverse situations. However, further research is needed to fully assess its long-term effectiveness and reliability as a component of dental education.

## Supplementary Information

Supplementary Material 1.

## Data Availability

The data analyzed during this study are not publicly accessible but can be obtained from the corresponding author on reasonable request.

## Abbreviations

SARS‑CoV‑2: severe acute respiratory syndrome-coronavirus-2
COVID: 19 Coronavirus disease 2019
