# Supplementary material for: The impact of preclinical education on operative and restorative skills in dental students: a survey-based evaluation
Source: BMC Med Educ. 2025 Nov 25;25:1647. doi: 10.1186/s12909-025-08252-x (PMC12648806; doi:10.1186/s12909-025-08252-x)
Supplement: Supplementary file 1 — Supplementary Material 1. [file 12909_2025_8252_MOESM1_ESM.docx]

**Appendix 1**

**Survey questionnaire**

Part A

1- Gender

2- Age

3- What grade were you in during the 2019-2020 academic year?

4- How did you receive your preclinical training?

Part B

1- I was able to detect occlusal and smooth surface caries.

2- I was able to detect approximal surface caries.

3- I used the hand instruments during treatment according to their intended purposes.

4- I used rotary instruments and burs for their intended purposes during the treatment.

5- I removed caries effectively.

6- I performed an ideal cavity preparation for an anterior composite resin restoration.

7- I performed an ideal cavity preparation for a posterior composite resin restoration.

8- I achieved ideal placement of the matrix and wedge in anterior interproximal cavities.

9- I achieved ideal placement of the matrix and wedge in posterior interproximal cavities.

10- I applied indirect and ergonomic working principles.

11- I applied liner materials according to their indications, guided by theoretical knowledge.

12- I applied base materials in accordance with their indications, guided by theoretical knowledge.

13- I accurately performed the clinical steps of adhesive system application.

14- I used the layering technique to properly apply composite resins into the cavity.

15- I completed the finishing and polishing procedures of the composite resin restorations ideally.

16- I ideally checked the occlusion and height at the end of the composite resin restorations.
